# *Notes from the Field:* Botulism Outbreak Associated with Home-Canned Peas — New York City, 2018

**DOI:** 10.15585/mmwr.mm6810a5

**Published:** 2019-03-15

**Authors:** Genevieve Bergeron, Julia Latash, Cherry-Ann Da Costa-Carter, Christina Egan, Faina Stavinsky, John Arek Kileci, Alison Winstead, Benyang Zhao, Michael J. Perry, Kevin Chatham-Stephens, Dost Sarpel, Scott Hughes, Maureen A. Conlon, Seth Edmunds, Mirna Mohanraj, Jennifer L. Rakeman, Dominick A. Centurioni, Carolina Lúquez, Amy K. Chiefari, Scott Harper

**Affiliations:** ^1^Epidemic Intelligence Service, CDC; ^2^Bureau of Communicable Disease, Division of Disease Control, New York City Department of Health and Mental Hygiene; ^3^Council of State and Territorial Epidemiologists/CDC Applied Epidemiology, Atlanta, Georgia; ^4^Public Health Laboratory, Division of Disease Control, New York City Department of Health and Mental Hygiene; ^5^Wadsworth Center, New York State Department of Health, New York, New York; ^6^Division of Environmental Health, New York City Department of Health and Mental Hygiene; ^7^Division of Pulmonary, Critical Care and Sleep Medicine, Mount Sinai St. Luke's, New York, New York; ^8^Division of Foodborne, Waterborne, and Environmental Diseases, CDC; ^9^Division of Infectious Diseases, Icahn School of Medicine at Mount Sinai, New York, New York; ^10^Oak Ridge Institute for Science and Education, Oak Ridge, Tennessee; ^11^Division of State and Local Readiness, CDC.

On June 6, 2018, at 1:30 p.m., the New York City Department of Health and Mental Hygiene was notified of three related women who had arrived at a hospital 4 hours earlier for evaluation for acute nausea, dizziness, blurred vision, slurred speech, ptosis, thick-feeling tongue, and shortness of breath. Two patients developed respiratory failure, requiring intubation and mechanical ventilation in the emergency department, and the third patient was intubated at 7 p.m. that evening. The combination of cranial nerve palsies and respiratory failure in multiple patients suggested botulism, a paralytic illness caused by botulinum neurotoxin (BoNT), most commonly produced by *Clostridium botulinum.*

 Approximately 14 hours before arriving at the hospital, the patients had shared a homemade potato salad containing home-canned peas. The family’s freezer had malfunctioned, and, to preserve some commercially produced frozen peas, one of the patients had home-canned the peas 1–2 weeks before consumption. After consultation with CDC (https://www.cdc.gov/botulism/health-professional.html), botulinum antitoxin was released by CDC and administered to all patients within approximately 12 hours of arrival at the hospital. All three patients survived but required prolonged intensive care (range = 34–54 days) and rehabilitation.

Blood specimens were collected from two patients before administration of antitoxin, and stool specimens were collected from all three after antitoxin administration. Testing included mouse bioassay at the New York City Department of Health and Mental Hygiene Public Health Laboratory and mass spectrometry, reverse transcription–polymerase chain reaction (RT-PCR), and culture at the New York State Department of Health Wadsworth Center. All three stool specimens tested positive by RT-PCR for BoNT type A with a silent B gene (BoNT type A(B)). A wash from the empty jar that previously held the peas and residual food from the salad bowl also tested positive by RT-PCR for BoNT type A(B). Whole genome sequencing demonstrated that the isolates recovered from two stool specimens were indistinguishable from the salad bowl isolate. Other environmental samples, including different home-canned vegetables from the same batch, were negative for BoNT, confirming that the peas were the outbreak source.

The patient who prepared the home-canned peas was a novice home canner. She used a peach preserves recipe with a boiling water technique, replacing the peaches with frozen vegetables. The patient was unaware that low-acid foods (e.g., vegetables) must be canned in a pressure canner rather than a boiling water canner to eliminate *C. botulinum* spores (*1*). After the jars cooled, the patient correctly checked for jar seal. One of the jars of peas was not sealed, so the patient covered and refrigerated it, and the family consumed the peas in the potato salad. The U.S. Department of Agriculture guidelines state that “foods in single unsealed jars could be stored in the refrigerator and consumed within several days” (*1*). However, this recommendation applies only to cans that have been correctly processed. In the absence of a pressure-canning step, *C. botulinum* spores were not eliminated, and the closed jar created an anaerobic environment allowing spore germination and BoNT production.

This outbreak illustrates the importance of educating home canners on safe home-canning practices to prevent botulism. Home-canned food, even when made with commercially processed ingredients, can lead to morbidity or mortality if canned incorrectly. Safe home-canning guidelines need to be followed (*1*), especially with low acidity foods, and when processing errors occur, foods should be discarded or reprocessed according to recommended guidelines within 24 hours.
